# Effects of use motivations and alexithymia on smartphone addiction: mediating role of insecure attachment

**DOI:** 10.3389/fpsyg.2023.1227931

**Published:** 2023-07-17

**Authors:** Xinquan Jin, Qiang Jiang, Weiyan Xiong, Wei Zhao

**Affiliations:** ^1^Jiangsu Research Center of “Internet Plus Education,” Jiangnan University, Wuxi, China; ^2^College of Information Science and Technology, Northeast Normal University, Changchun, China; ^3^Department of International Education, Education University of Hong Kong, Hong Kong, Hong Kong SAR, China

**Keywords:** smartphone addiction, use motivations, alexithymia, insecure attachment, adolescents

## Abstract

**Background and objectives:**

Concern exists regarding the potential negative consequences of smartphone addiction among adolescents. This study investigated the effect of use motivations and alexithymia on smartphone addiction among adolescents with two insecure attachment styles, namely, anxious and avoidant attachment. These attachment styles were regarded as mediating variables.

**Methods:**

Self-report measures were used to assess use motivations, alexithymia, insecure attachment and smartphone addiction. Data were collected from 748 junior high school students (382 males and 366 females) in northeastern China. Structural equation modeling was used to test our hypothesis that use motivations and alexithymia are directly associated with smartphone addiction and also through the mediation of attachment insecurities.

**Results:**

The structural equation modeling results showed a strong and positive correlation between use motivation and smartphone addiction, with avoidant attachment mediating such a relationship. Meanwhile, the two components of alexithymia, difficulty identifying feelings and externally oriented thinking, positively predicted smartphone addiction, with avoidant attachment serving as a mediator of this effect. In addition, the mediation analysis results demonstrated that attachment anxiety mediated the connection between escape drive, extrinsically focused thought, and smartphone addiction.

**Conclusion:**

Findings describe how attachment insecurities, smartphone use motivations, and alexithymia can interact with one another to predict smartphone addiction. Smartphone use motivation types and alexithymia symptoms should be taken into consideration when designing targeted intervention programs for smartphone addiction to address the different attachment needs of adolescents, which would be helpful to reduce their smartphone addiction behaviors.

## 1. Introduction

Smartphone addiction is a behavioral disorder demonstrated by people with extreme dependence on their smartphones, negatively affecting not only their health but also their psychological and social aspects (Li et al., 2021; Gao et al., 2022; Lai et al., 2022). Accordingly, the factors that lead to smartphone addiction, including family dysfunction, experiential avoidance, and social motivation for smartphones, have attracted extensive scholarly attention (Liu et al., 2020; Chatterjee et al., 2022). However, only a few scholars have explored the factors that affect smartphone addiction in adolescents and the effects of personal factors and affective factors on smartphone addiction are not isolated (Busch and McCarthy, 2021; Huang et al., 2021). A holistic picture of these factors is yet to be developed to formulate effective solutions to the problem. Therefore, exploring the potential antecedent variables that can effectively predict smartphone addiction among adolescents has become an urgent matter.

These predictive factors have attracted wide research attention given the prevalence of smartphone use among adolescents (Ng and Chan, 2020; An et al., 2022). Introduced by Brand et al. (2019), the Interaction of Person-Affect-Cognition-Execution (I-PACE) model has been recently adopted to understand the reasons behind the smartphone addiction behaviors of adolescents, and results point toward a correlation between addictive human behaviors and affective responses stimulated by personality traits. A growing body of research has been dedicated to examining the relationships of individual factors to behavioral addictions, such as motivations and alexithymia (Gao et al., 2018; Gündoğmuş et al., 2021), and to exploring perceptions of executive dysfunction (Ge et al., 2022), and fear of miss out (Bajwa et al., 2022) as mediating mechanisms of these relationships. Besides, the risk of developing smartphone addiction has been shown to be higher among adolescents with insecure attachment styles (Xiao et al., 2021). Surprisingly, no empirical studies have directly examined the possible mediating role of insecure attachment styles in the connections between addictive behaviors and personality traits (Li et al., 2022). Although these studies have made advances, most have only concerned taking the insecure attachment or alexithymia variables as a whole, which is insufficient to explain people’s varying sensitivities to smartphone addiction (Liu and Ma, 2019; Ding et al., 2022). However, most of the previous studies on the mediation between these variables have been done on college students, with less attention to adolescents. Therefore, the mediating role of attachment insecurities warrants further study to understand the mechanisms behind adolescents’ smartphone addiction clearly, use motivations, alexithymia, and insecure attachment.

In the transition from smartphone usage to addiction, the personal motivations for use play a significant role in the behavior of adolescents (Fu et al., 2020; Chen et al., 2023). Motivation is the process of eliciting and sustaining goal-directed behavior (Dong et al., 2017; Schaller et al., 2017). Many studies have explored the relationship between use motivations and smartphone addiction due to the central role of smartphone use in the emergence of addiction behavior (Wen et al., 2022). Smartphones are initially used by individuals to engage in social interactions (Li et al., 2021). In this case, a common motivator for people to use smartphones is social motivation. Furthermore, people also tend to use their smartphones when facing certain issues as a way to escape their reality or to seek the advice or accompaniment of others (Zhang and Wu, 2022). This type of motivator can be described as escape motivation. According to the uses and gratification perspective, adolescents attempt to satisfy psychological requirements by excessively using their smartphones (Eighmey and McCord, 1998), but doing so can shape their behaviors and motivate their continuous usage of these devices (Zhang et al., 2022). Based on the above review, the following hypotheses are proposed:

H1: Smartphone addiction is positively affected by escape motivation.

H2: Smartphone addiction is positively affected by social motivation.

Furthermore, alexithymia is another predisposing factor considered to increase the risk of smartphone addiction. Alexithymia is a portmanteau of the Greek words a (no), lexis (word), and timia (affection), which, when taken together, refer to having “no words for feelings” (Hassen et al., 2023). This relatively stable personality trait represents a cluster of characteristics that reflect the challenges faced by individuals when they are processing feelings at the affective and cognitive levels. Alexithymia mainly includes difficulty identifying feelings (DIF), difficulty describing feelings (DDF), and externally oriented thinking (EOT) (Sifneos, 1973). As a multifaceted personality construct, there is clear evidence that alexithymia has been associated with smartphone addiction, but this relationship has different effects across the three dimensions (Ghorbani et al., 2017; Sutherland et al., 2022). For instance, Dalbudak et al. (2013) demonstrated a link between DIF and DDF, two dimensions of alexithymia, and Internet addiction. Inversely, the EOT of alexithymia would be less or not at all related to addiction behavior. However, in other work, only EOT was associated with addictive behavior (Ghorbani et al., 2017; Zhang et al., 2022). Indeed, Ng and Chan (2020) found that alexithymia affects around 36% of adolescents in China, and individuals with alexithymia often show stereotyped emotions that influence their decision making. More specifically, individuals who have difficulty in identifying and describing feelings are more likely to exhibit problems with emotional self-regulation, in which emotional distress is often associated with problematic behaviors (Nigro et al., 2017; Hao and Jin, 2020). Moreover, prior research has suggested that adolescents, contrary to adults who are better able to regulate their emotions, have a tendency to suppress or stifle their feelings and are easily overwhelmed by negative emotions (Zhou et al., 2022). This may lead them to perceive smartphones as an excellent tool for them to escape reality, hide their emotions, and avoid showing emotional responses (Fu et al., 2020). Such perception only exacerbates their excessive dependence on these devices. On the basis of these arguments, the following hypotheses regarding the components of alexithymia are proposed:

H3: Smartphone addiction is positively affected by DIF.

H4: Smartphone addiction is positively affected by DDF.

H5: Smartphone addiction is positively affected by EOT.

Based on the I-PACE model, adolescent addictive behavior may be influenced not only by predisposing personality factors but also by their interactions with insecure attachment. According to attachment theory, attachment can be understood as a deep, enduring emotional bond that reflects an individual’s propensity to feel secure or insecure in his/her relationship with his/her object of attachment (Bowlby, 1969). Attachment insecurities have two dimensions, namely, attachment avoidance and attachment anxiety (Bowlby, 1973), of which the former describes the fear of being abandoned, whereas the latter describes the fear of losing one’s independence by becoming excessively dependent on others (Bowlby, 1969, 1973). Studies with adolescents have revealed positive associations between smartphone addiction and avoidance/anxiety attachment. A person lacking secure attachments may suffer from relational insecurity along with loss of control, obvious cravings, or unregulated emotions, all of which can increase his/her risk of developing addictive behavior, including smartphone addiction (Kim and Koh, 2018; Gao et al., 2022; Chen et al., 2023). Those individuals who are prone to having an avoidant attachment style rarely express their need for affection or care and thus tend to reject help or emotional support from others for a sense of personal independence and freedom (Fraley et al., 2011). Accordingly, Musetti et al. (2022) established a link between higher levels of smartphone addiction and attachment avoidance. Other studies also suggest that people with high levels of attachment anxiety tend to experience smartphone addiction. For instance, Wolniewicz et al. (2020) found that people with high attachment-related anxiety compulsively demand others to offer them their support just for them to feel loved. These people prefer interacting with others both selectively and autonomously as allowed by their smartphones. This finding alludes to the notion that adolescents with attachment anxiety regularly use their smartphones to track the whereabouts or activities of their loved ones (Chatterjee et al., 2022), and such behavior ultimately leads to smartphone addiction. Following these arguments, the following hypotheses are proposed:

H6: Smartphone addiction is positively affected by attachment avoidance.

H7: Smartphone addiction is positively affected by attachment anxiety.

Although insecure attachment has been shown to have a positive effect on smartphone addiction, it is unclear whether two types of insecure attachment styles, attachment avoidance and attachment anxiety, are affected by motivations to use smartphones and alexithymia. On the one hand, previous studies have offered useful insights into the correlation between use motivations and attachment insecurities (Liese et al., 2020). According to the displacement hypothesis, computerized media such as smartphones reduce the strength of social relationships by allowing people to interact without being face-to-face (Jauregui and Estevez, 2020). In other words, consciously using a smartphone to meet one’s psychological needs, whether socially or escape motivated, will reduce one’s time to engage in interpersonal communication, thereby compromising secure attachment (Fu et al., 2020). On the other hand, several studies have investigated the relationship between insecure attachment and alexithymia, but the results have been inconsistent (Musetti et al., 2022). Most studies have pointed out that insecure attachment refers to a disordered “internal working model.” Those who are burdened with insecure attachments adopt maladaptive emotional strategies that lead to increased levels of alexithymia (Kim et al., 2017; Yuchang et al., 2017). However, Tarantino et al. (2018) discovered that adolescence is a crucial transitional period during which developing attachment patterns is a critical developmental task. This process does not occur in isolation, but rather in the context of close and enduring relationships with others. Consequently, adolescents with alexithymia are at a higher risk of developing insecure attachment styles (Besharat et al., 2014; Scigala et al., 2021). According to I-PACE, disordered characteristics of persons tend to shape associated affective and addictive behavioral responses. In this direction, adolescents with insecure attachment may be affected by difficulties in emotion regulation in alexithymia and are associated with smartphone addiction (Pellerone et al., 2017; Lyvers et al., 2022). Boldrini et al. (2021) and Scigala et al. (2022) found proof to support the relationships of alexithymia with both attachment anxiety and attachment avoidance, which could be ascribed to the challenges faced by individuals when regulating their emotions. Other scholars have argued that due to their lack of psychosocial resources, such as high cognitive impairment and limited social support, individuals with alexithymia tend to alleviate their negative feelings via attachment avoidance or anxiety coping mechanisms, which lead to attachment insecurities (Xiang et al., 2022). The hypotheses are as follows:

H8: Attachment avoidance is positively affected by escape motivation (H8a) and social motivation (H8b).

H9: Attachment anxiety is positively affected by escape motivation (H9a) and social motivation (H9b).

H10: Attachment avoidance is positively influenced by DIF (H10a), DDF (H10b), and EOT (H10c).

H11: Attachment anxiety is positively influenced by DIF (H11a), DDF (H11b), and EOT (H11c).

**FIGURE 1:**
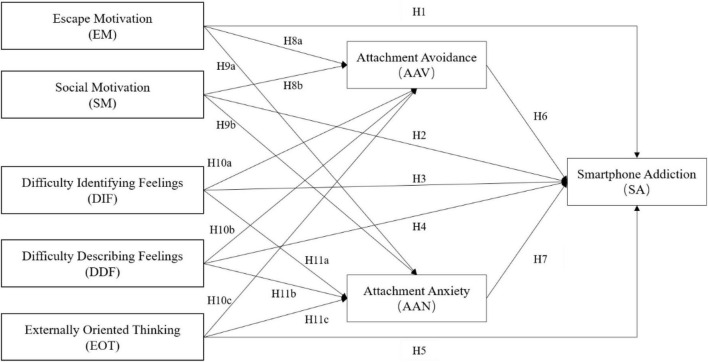
Hypothesized research model.

Therefore, in consideration of the above, this study was conducted to explore the mediating effects of attachment insecurities in the relationship between smartphone addiction with use motivations and alexithymia. Figure 1 illustrates the proposed model based on the above hypotheses.

## 2. Materials and methods

### 2.1. Participants

Convenience sampling was adopted to recruit participants from junior high schools in Northeast China. This study followed and met the ethical requirements of the researchers’ institutions. A total of 856 online questionnaires were completed by Chinese junior school students, whom, along with their parents, gave their informed consents to participate in the study. Among the collected responses, 748 were kept for analysis (87.38% effective rate) after excluding those questionnaires that were deemed invalid for various reasons (e.g., choosing the same response for all questions or leaving too many missing values). A total of 382 (51.07%) male and 366 (48.93%) female participants comprised the sample. These participants were aged between 12 and 15 years (*M* = 12.86; SD = 0.77). In terms of educational level, those participants in grades 7, 8, and 9 comprised 32.62% (244), 38.77% (290), and 28.61% (214) of the sample, respectively. In terms of their smartphone use duration, 38 (5.08%), 181 (24.20%), 386 (51.60%), 128 (17.11%), and 15 (2.01%) participants admitted using their smartphones for less than an hour, 1 to 3 h, 4 to 6 h, 7 to 9 h, and more than 10 h each day, respectively.

### 2.2. Instrumentation

This study adopted four scales for the data collection, namely, the Smartphone Addiction Inventory-Short Form (SPAI-SF) to categorize the smartphone addiction levels of the participants, the Smartphone Use Motivations Scale to determine the motivations behind their smartphone use, the Toronto Alexithymia Scale (TAS-20) to measure their alexithymia attributes, and the Relationship Structures Questionnaire (RSQ) to gauge their insecure attachment. The researchers also developed a Personal Information Form to collect the demographic data of the participants. These instruments, the validity and reliability of which have been proven in the literature, were utilized without any modification.

Developed by Lin et al. (2017), SPAI-SF contains ten items that evaluate the propensity of adolescents to develop smartphone addiction behavior. A sample item is “I try to spend less time on smartphones, but the efforts were in vain.” Each item is rated on a four-point Likert scale (“strongly disagree” to “strongly agree”). SPAI-SF has a composite score ranging from 10 to 40, with a higher score corresponding to a higher risk of developing smartphone addiction behavior. The SPAI-SF has been validated among Chinese adolescents and has demonstrated good internal consistency (Cronbach’s alpha = 0.77–0.81; Zhang et al., 2023). In this study, this scale reported an internal consistency of 0.889.

The six-item Smartphone Use Motivations Scale was developed based on the results of previous studies on the motivations for smartphone use (Leung and Wei, 2000; Li et al., 2021) to evaluate the escape motivation of smartphone users. A sample item is “It makes me feel less alone.” Each item is measured on a seven-point Likert scale (“strongly disagree” to “strongly agree”). This scale has a Cronbach’s alpha of 0.82. Five items for measuring social motivation, such as “I use a smartphone to socialize with others,” were adapted from the Smartphone Use Motivations Scale developed by Dong et al. (2017). The Smartphone Use Motivations Scale has also been proven to have strong reliability and validity among Chinese adolescents (Cronbach’s alpha = 0.781). In this study, the questions for escape and social interaction motivation obtained Cronbach’s alpha coefficients of 0.852 and 0.857, respectively.

Developed by Bagby et al. (1994) and adapted into Chinese by Zhu et al. (2007), TAS-20 is a 20-item scale for measuring alexithymia. Each item is measured on a five-point Likert scale (“strongly disagree” to “strongly agree”). TAS-20 is divided into the three sub-dimensions of DIF (e.g., “I often don’t know why I am angry.”), DDF (e.g., “I find it hard to describe how I feel about people.”), and EOT (e.g., “I prefer to just let things happen rather than to understand why they turned out that way.”). A higher composite score from these sub-dimensions corresponds to a higher degree of alexithymia. In the initial study, this scale has an overall Cronbach’s alpha of 0.87, whereas its sub-dimensions have internal consistency coefficients ranging from 0.65 to 0.80. In this study, these sub-dimensions reported an overall Cronbach’s alpha of 0.83.

Developed by Fraley et al. (2011) and adapted into Chinese by Zhang et al. (2022), the RSQ of the Experiences in Close Relationships-Revised (ECR-RS) contains nine items that evaluate the attachment styles of adolescents. These items offer prototypical descriptions of avoidance (6 items) and anxiety (3 items), with each item measured on a seven-point Likert scale (“strongly disagree” to “strongly agree”) to indicate the participants’ degree of intimacy with their parents. Sample items are “I worry a lot about my relationships” (attachment anxiety) and “I am nervous when parents get too close to me” (attachment avoidance). In Zhang et al. (2022), this scale obtained internal consistency coefficients ranging from 0.71 to 0.80. However, in this study, attachment avoidance and anxiety obtained Cronbach’s alpha values of 0.904 and 0.867, respectively.

### 2.3. Procedure

The data were collected between November and December 2022 using sojump.com, an online survey platform in China. The participants submitted their voluntary participation forms, including their and their parents’ consent forms, prior to the survey. All participants were informed that the collected data would be kept confidential and would only be available to the researchers. The links to the Online Parent Consent Forms were sent to the participants via their social media accounts, and the link to the online survey was sent once receiving the signed consent form.

### 2.4. Analysis procedures

The relationships among use motivations, alexithymia, insecure attachment, and smartphone addiction were analyzed by inputting the collected data into SPSS 21 and AMOS 26. A descriptive analysis of the data was then conducted, followed by a validity check of the adopted instruments. The normally distributed data were processed using AMOS 26 for the mediation analysis and to evaluate both the measurement and structural models. Following the suggestions of Schreiber et al. (2006), the overall fit was assessed using several indices, including chi-square (χ^2^), χ^2^/df ratio, incremental fit index (IFI), comparative fit index (CFI), Tucker-Lewis index (TLI), and root mean square error of approximation (RMSEA) (Browne and Cudeck, 1989). A model is said to have an acceptable fit when its CFI and TLI values are greater than or equal to 0.80 and its RMSEA values are less than or equal to 0.08 (Schreiber et al., 2006).

## 3. Results

### 3.1. Preliminary analyses

The skewness and kurtosis of the data were initially evaluated to ensure the trustworthiness of the dataset. The sample had a nearly normal distribution as reflected in the skewness (−1.67 to 0.51) and kurtosis (−1.25 to 3.00) of all variables, which were within acceptable ranges (<2 for skewness and <4 for kurtosis) (Kline, 2005). Descriptive statistics for study variables are presented in Table 1. And none of these variables showed any significant association with gender (*p* > 0.05).

**TABLE 1:** Descriptive statistics.

Scale	Females		Males		M	SD	t	*p*
	**M**	**SD**	**M**	**SD**				
Smartphone addiction (SA)	30.24	8.83	31.37	9.35	30.82	9.11	-1.698	0.090
Difficulty identifying feelings (DIF)	21.23	5.81	21.15	5.59	21.19	5.69	0.193	0.847
Difficulty describing feelings (DDF)	16.20	3.90	15.80	4.17	16.00	4.04	1.348	0.178
Externally oriented thinking (EOT)	23.08	5.97	23.75	6.29	23.42	6.14	-1.504	0.133
Social motivation (SM)	13.19	4.57	13.14	4.37	13.17	4.46	0.161	0.872
Escape motivation (EM)	16.90	3.20	16.92	3.28	16.91	3.23	-0.085	0.933
Attachment avoidance (AAV)	3.59	1.33	3.62	1.34	3.60	1.33	-0.242	0.809
Attachment anxiety (AAN)	4.20	1.45	4.12	1.52	4.16	1.48	0.678	0.498

### 3.2. Evaluation of the measurement model

As indicated by a Kaiser-Meyer-Olkin value of 0.91 and Bartlett’s test of sphericity value of 19428.108 (*p* < 0.001), these 50 items were sufficiently correlated to warrant a component analysis. Given their poor factor loadings, items SM3 and SM4 in social motivation and EM2 and EM3 in escape motivation were removed. Moreover, one item of the escape motivation variable (EM6) had multiple loadings on both factors and was thus excluded from further analysis. Then, the eight factors obtained Cronbach’s alpha coefficients ranging from 0.75 to 0.92, which exceeded the 0.70 thresholds (Sanchez, 2013).

After rotation, all items obtained statistically significant (*p* < 0.001) factor loadings ranging from 0.61 to 0.91. Supplementary Table 1 presents the confirmatory factor analysis (CFA) results. The factor loadings of all variables exceeded 0.7. Meanwhile, their average variance extracted (AVE) and CR values exceeded 0.511 and 0.715, respectively, which are above their respective thresholds (0.5 and 0.7). These results confirm the sufficient reliability and convergent validity of the selected factors. Moreover, as shown in Table 2, the structural equation modeling (SEM) correlation coefficients of these dimensions were below their corresponding squared AVEs, thus indicating that the selected factors have satisfactory discriminant validity.

**TABLE 2:** Results of the measurement model.

	Reliability		AVE	Correlation coefficients							
	**α**	**CR**		**1**	**2**	**3**	**4**	**5**	**6**	**7**	**8**
1. SA	0.889	0.912	0.511	**0.715**							
2. DIF	0.914	0.914	0.570	0.314***	**0.753**						
3. DDF	0.901	0.901	0.567	0.219***	0.092***	**0.733**					
4. EOT	0.857	0.848	0.537	0.421***	0.239***	0.185***	**0.755**				
5. SM	0.852	0.853	0.659	0.525***	0.233***	0.171***	0.285***	**0.812**			
6. EM	0.873	0.873	0.697	0.216***	0.198***	0.079***	0.182***	0.125***	**0.835**		
7. AAV	0.904	0.901	0.606	0.39***	0.194***	0.285***	0.282***	0.273***	0.176***	**0.779**	
8. AAN	0.867	0.871	0.694	0.319***	0.150***	0.102***	0.204***	0.238***	0.140***	0.163***	**0.833**

The squared AVE values are written in boldface. ***p < 0.001. α, Cronbach’s alpha reliability coefficient; SA, smartphone addiction; DIF, difficulty identifying feelings; DDF, difficulty describing feelings; EOT, externally oriented thinking; SM, social motivation; EM, escape motivation; AAV, attachment avoidance; AAN, attachment anxiety.

### 3.3. Test of the structural model and hypothesis testing

**FIGURE 2:**
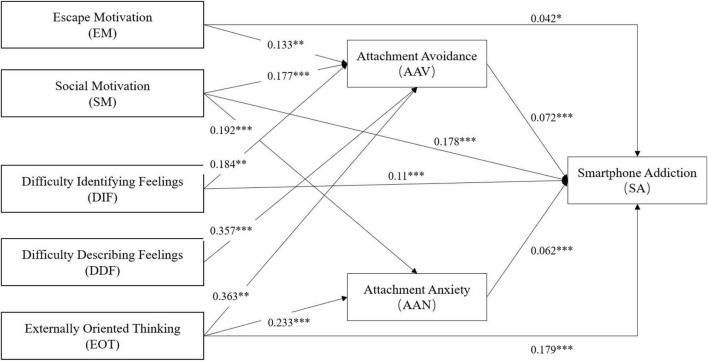
Final research model. *p < 0.05; **p < 0.01; ***p < 0.001.

Structural equation modeling was performed using AMOS to test the hypotheses, and the resulting path model (Figure 2 and Table 3) reported a good fit (χ^2^/df = 2.729, IFI = 0.916, CFI = 0.916, TLI = 0.911, RMSEA = 0.048). Among the 17 hypotheses, only 13 were supported as the coefficient beta for the respective hypothesized paths reached statistical significance.

**TABLE 3:** Hypothesis testing results.

Hypotheses	Path	Estimate	S.E.	C.R.	*p*	Results
H1	SM→SA	0.178	0.017	10.215	0.027*	Supported
H2	EM→SA	0.042	0.019	2.214	***	Supported
H3	DIF→SA	0.110	0.026	4.229	***	Supported
H4	DDF→SA	0.033	0.021	1.561	0.118	Not Supported
H5	EOT→SA	0.179	0.027	6.727	***	Supported
H6	AAV→SA	0.072	0.015	4.768	***	Supported
H7	AAN→SA	0.062	0.015	4.061	***	Supported
H8a	EM→AAV	0.133	0.051	2.600	0.009**	Supported
H8b	SM→AAV	0.177	0.040	4.439	***	Supported
H9a	EM→AAN	0.106	0.052	2.029	0.056	Not Supported
H9b	SM→AAN	0.192	0.041	4.684	***	Supported
H10a	DIF→AAV	0.184	0.070	2.636	0.008**	Supported
H10b	DDF→AAV	0.357	0.057	6.253	***	Supported
H10c	EOT→AAV	0.363	0.068	5.367	***	Supported
H11a	DIF→AAN	0.108	0.071	1.522	0.128	Not Supported
H11b	DDF→AAN	0.069	0.057	1.203	0.229	Not Supported
H11c	EOT→AAN	0.233	0.068	3.395	***	Supported

*p < 0.05; **p < 0.01; ***p < 0.001. SA, smartphone addiction; DIF, difficulty identifying feelings; DDF, difficulty describing feelings; EOT, externally oriented thinking; SM, social motivation; EM, escape motivation; AAV, attachment avoidance; AAN, attachment anxiety.

Five factors, namely, EM (β = 0.042; *p* < 0.05), SM (β = 0.178; *p* < 0.001), DIF (β = 0.11; *p* < 0.001), EOT (β = 0.179; *p* < 0.001), AAV (β = 0.072; *p* < 0.05), and AAN (β = 0.062; *p* < 0.001) were significantly and positively associated to smartphone addiction, thus supporting hypotheses H1 to H3 and hypotheses H5 and H7. However, smartphone addiction was not significantly related to DDF (β = 0.033; *p* = 0.118 > 0.05). The effects of EM (β = 0.133; *p* < 0.01), SM (β = 0.177; *p* < 0.001), DIF (β = 0.184; *p* < 0.01), DDF (β = 0.357; *p* < 0.001), and EOT (β = 0.363; *p* < 0.001) on AAV were also confirmed, thus supporting hypotheses H8a and H8b and hypotheses H10a to H10c. Meanwhile, ANN was confirmed to be influenced by both SM (β = 0.192; *p* < 0.001) and EOT (β = 0.233; *p* < 0.001), thus supporting hypotheses H9b and H11c. However, ANN was not significantly affected by DIF (β = 0.108; *p* = 0.128 > 0.05) and DDF (β = 0.069; *p* = 0.229 > 0.05), thus rejecting hypotheses H11a and H11b.

### 3.4. Test of the mediation effect

Considering the insignificant path coefficients among EM, DDF, DIF and AAN, and between DDF and SA, the mediating influence of AAV on the relationship among SM, EM, DIF, EOT, and SA was tested along with that of AAN on the relationship among SM, EOT, and SA. AMOS was used to simulate the bootstrapped 95% confidence intervals (CI) with 5,000 bias corrections, as shown in Table 4. Since 95% confidence intervals (95% CI) did not comprise zero, the relationships of smartphone addiction with DIF [indirect effect = 0.013, 95% CI (0.004, 0.028), *p* < 0.01], EOT [indirect effect = 0.026, 95% CI (0.014, 0.047), *p* < 0.01], EM [indirect effect = 0.001, 95% CI (0.003, 0.020), *p* < 0.001], and SM [indirect effect = 0.013, 95% CI (0.006, 0.023), *p* < 0.01] were significantly mediated by AAV, while its relationships with SM [indirect effect = 0.012, 95% CI (0.005, 0.021), *p* < 0.01] and EOT [indirect effect = 0.014, 95% CI (0.005, 0.028), *p* < 0.01] were mediated by AAN.

**TABLE 4:** Mediation analysis results.

Tested relationship	Direct effect	Indirect effect	Total effect	95% CI
EM = > AAV = > SA	0.042*	0.01**	0.068**	0.003–0.020
SM = > AAV = > SA	0.178**	0.013**	0.191*	0.006–0.023
SM = > AAN = > SA	0.178**	0.012**	0.190**	0.005–0.021
DIF = > AAV = > SA	0.110**	0.013**	0.123**	0.004–0.028
EOT = > AAV = > SA	0.179**	0.026**	0.205**	0.014–0.047
EOT = > AAN = > SA	0.179**	0.014**	0.205**	0.005–0.028

*p < 0.05; **p < 0.01. SA, smartphone addiction; DIF, difficulty identifying feelings; DDF, difficulty describing feelings; EOT, externally oriented thinking; SM, social motivation; EM, escape motivation; AAV, attachment avoidance; AAN, attachment anxiety.

## 4. Discussion

This study aims to investigate those factors that drive the smartphone addiction behavior of Chinese adolescent students. Findings suggested that attachment avoidance mediated the relationship between use motivations, DIF and EOT with smartphone addiction, while attachment anxiety only mediated the relationship between escape motivation and EOT with smartphone addiction.

### 4.1. Interpretations of main findings

First, the results for the relationships between alexithymia traits and insecure attachment styles were in line with those obtained by Scigala et al. (2022). Moreover, consistent with the findings of Xiao et al. (2021), smartphone addiction was positively predicted by DIF and EOT. While supporting the findings of Ridout et al. (2021), smartphone addiction did not show any significant correlation with the DDF subscales of TAS-20, hence rejecting the research hypotheses. This result could be ascribed to the relationship of DDF with mindfulness facets, leading to less contact with other people. According to Scigala et al. (2021), individuals exhibiting elevated levels of DDF might be perceived as distant or antagonistic by their peers. Despite this, they tend to resist conforming to external expectations, resulting in unfavorable responses from others. Notably, it has been shown that mindfulness facets negatively mediated the relationship between alexithymia and smartphone addiction (Yuchang et al., 2017). As a result, individuals who have difficulty describing their feelings would act with self-awareness and also show a tendency toward less connection with others, which may lead to less frequent smartphone use. As mentioned earlier, having both DIF and EOT can lead to challenges in emotion regulation, thus increasing the tendency for individuals to adopt unhealthy coping strategies, and eventually trigger smartphone addiction (Zhou et al., 2022).

Second, consistent with previous research (e.g., Kim and Koh, 2018; Zhang and Wu, 2022), smartphone addiction was predicted by motivations for using smartphones. Although smartphone use among adolescents is driven by different motivations, their motivations for internal escape and external social interaction are internal psychological factors that depend on their individual needs and define the recurrence of targeted behaviors in their daily lives (Schaller et al., 2017). According to Zhang et al. (2022), an individual’s level of motivation to use their smartphone is directly linked to the frequency and duration of smartphone usage, which ultimately increases the risk of developing smartphone addiction. Therefore, personal needs define the smartphone usage behavior of individuals (Eighmey and McCord, 1998; Busch and McCarthy, 2021). Those adolescents that frequently satisfy their personal motivation by using their smartphones are more likely to become overdependent on these devices (Wen et al., 2022).

Third, this study incorporates insecure attachment styles (i.e., attachment avoidance and attachment anxiety) as a mediating variable to explore the potential mechanisms that underlie the relationship among alexithymia, smartphone use motivations, and smartphone addiction. Consistent with our assumption, attachment avoidance is confirmed to mediate the association of smartphone addiction with several factors, including DIF, EOT, escape motivation and social motivation. People showing attachment avoidance tend to have the interpersonal functioning of avoidance and apathy; therefore, these people tend to leave their feelings unexplored and not care about the feelings of others (Jauregui and Estevez, 2020; Ghinassi and Casale, 2023). Other scholars have shown that users with smartphone addiction behavior consider their smartphone as an instrument that helps them escape reality (Boldrini et al., 2021; Gao et al., 2022). Therefore, one may argue that the tendency for an individual to become addicted to smartphones depends on the strength of his/her avoidance attachment. The findings of this study also highlight the mediating effect of attachment anxiety on the relationships between smartphone addiction with EOT and social motivation. However, this variable does not mediate the relationships of smartphone addiction with DIF, and escape motivation. This finding can be attributed to the fact that attachment anxiety pertains to an individual’s intense desire to acquire and sustain the attention of others (Zhou et al., 2022). In contrast, people with DIF tend to experience difficulties with social interaction, avoid intimacy, and reject the idea of seeking help or closeness from others (Fraley et al., 2011; Kim and Koh, 2018). As a result, they display a greater degree of attachment avoidance rather than attachment anxiety. Additionally, the direct path from an avoidance motivation to attachment anxiety was less significant among individuals who use their smartphones to escape reality because of their greater desire to limit their contact with others and their tendency to avoid frustrations associated with social belonging (Fu et al., 2020; Musetti et al., 2022).

### 4.2. Theoretical and practical implications

This study contributes to the literature in two ways. First, this study offers a highly nuanced discussion of the relationship among smartphone addiction, alexithymia, use motivations, and insecure attachment for adolescents. While previous studies of smartphone addiction among adolescents have mostly focused on a single category of predictors (Busch and McCarthy, 2021), this work comprehensively explores the relationship of smartphone addiction with multiple factors. Specifically, results show that the increasing prevalence of smartphone addiction among adolescents is related to two types of motivation, three dimensions of alexithymia, and two types of insecure attachment. Second, consistent with the I-PACE model (Brand et al., 2019), the current study reveals that alexithymia and use motivations are influenced differently by attachment insecurities. While earlier studies have examined the overall role of various dimensions, a clear explanation of the influence of each variable is lacking as these studies have ignored the sub-dimensions (Xiao et al., 2021). By incorporating the effects of anxiety and avoidance attachment, smartphone addiction, and use motivations, the findings of this work offer a groundwork for further explorations of the mechanisms that underlie the development of smartphone addiction. In response to the increasing number of scholars that use the I-PACE model to understand smartphone addiction (Chatterjee et al., 2022; Wen et al., 2022), this study opens up a new avenue for investigating the mechanisms by which smartphone addiction is predicted by various personal and affective factors.

For practical implications, the findings of this work are supported by the I-PACE model, which suggests that smartphone addiction is directly influenced by attachment insecurities, use motivations, DIF, and EOT. Parents and teachers alike should determine how adolescents can be guided in regulating their negative feelings and balancing their interactions with the virtual and real worlds (Eighmey and McCord, 1998; Boldrini et al., 2021). Adolescents need to be taught how to interact positively with their real environments; moreover, they need to be provided an atmosphere where emotional support is readily available, especially at times when they face challenges (Chen, 2019; Shutzman and Gershy, 2023). Parents and teachers should also strive toward closing the emotional gap between them and adolescents by designing additional interventions that can compensate for the unmet needs of the latter, such as team counseling and leisure activities (Xiang et al., 2022). They also need to identify those adolescents with narrative disorders and insecure attachment styles in a timely manner and offer them much-needed support accordingly (Scigala et al., 2022).

### 4.3. Limitations and future studies

This study is not without its limitations. First, this work is limited by subjective self-report questionnaires (Zhang and Wu, 2022). Future studies can further validate the findings of this work by utilizing more objective techniques, such as smartphone apps, to monitor smartphone usage analysis and thus minimize reporting bias (Busch and McCarthy, 2021). Second, as our data were cross-sectional, it may limit the causal inference (Yuchang et al., 2019). To further validate the potential temporal association between each smartphone addiction and different variables among adolescents, future studies can consider conducting a longer follow-up period through longitudinal studies. Third, the mediators or outcome variables in this study were not related to the demographic variables of the participants and hence may affect the reliability of the findings regarding smartphone addiction among adolescents (Chen et al., 2023). Future studies can address this gap by collecting more relevant demographic information, such as family income and parents’ educational background, and adopting a larger sample size. Furthermore, the intricate correlations among the research variables were explored in this study using SEM. However, the SEM results could not be extrapolated to causality due to the cross-sectional nature of this research (Schaller et al., 2017). Future research can further validate the findings of this work by using longitudinal observations to understand how the development of smartphone addiction can be predicted by specific variables.

## 5. Conclusion

This study explored how smartphone addiction is influenced by alexithymia, use motivations and insecure attachment. Two types of insecure attachment, attachment avoidance and attachment anxiety, emerged as vital factors in facilitating smartphone addiction. Motivation positively influenced both smartphone addiction and insecure attachment and social motivation positively influenced attachment anxiety. Smartphone addiction among students was facilitated by DIF and EOT, of which only the latter had positive effects on attachment anxiety. Educators need to use more specific measures according to the different types of insecure attachment, use motivations, and alexithymia, to provide tailored interventions, which can alleviate smartphone addiction among adolescents and consequently improve their physical and mental health.

## Data availability statement

The original contributions presented in this study are included in this article/Supplementary material, further inquiries can be directed to the corresponding author.

## Ethics statement

The studies involving human participants were reviewed and approved by the Northeast Normal University. Written informed consent to participate in this study was provided by the participants’ legal guardian/next of kin.

## Author contributions

XJ: conceptualization, data analysis, writing-original draft preparation, and writing-review and editing. QJ: conceptualization, methodology, and writing-review and editing. WX: writing-review and editing and supervision. WZ: conceptualization, data collection, and formal analysis. All authors contributed to the article and approved the submitted version.

## References

[B1] An X. Chen S. Zhu L. Jiang C. (2022). The mobile phone addiction index: cross gender measurement invariance in adolescents. *Front. Psychol.* 13:894121. 10.3389/fpsyg.2022.894121 35923732 PMC9340052

[B2] Bagby R. M. Parker J. D. Taylor G. J. (1994). The twenty-item Toronto Alexithymia Scale-I. item selection and cross-validation of the factor structure. *J. Psychosom. Res.* 38 23–32. 10.1016/0022-3999(94)90005-1 8126686

[B3] Bajwa R. S. Abdullah H. Zaremohzzabieh Z. Jaafar W. M. W. Samah A. A. (2022). Smartphone addiction and phubbing behavior among university students: a moderated mediation model by fear of missing out, social comparison, and loneliness. *Front. Psychol.* 13:1072551. 10.3389/fpsyg.2022.1072551 36687837 PMC9853171

[B4] Besharat M. A. Naghshineh N. Ganji P. Tavalaeyan F. (2014). The moderating role of attachment styles on the relationship of alexithymia and fear of intimacy with marital satisfaction. *Int. J. Psychol. Stud.* 6 106–117. 10.5539/ijps.v6n3p106

[B5] Boldrini T. Mancinelli E. Erbuto D. Lingiardi V. Muzi L. Pompili M. (2021). Affective temperaments and depressive symptoms: the mediating role of attachment. *J. Affect. Disord.* 293 476–483. 10.1016/j.jad.2021.06.026 34256209

[B6] Bowlby J. (1969). *Attachment and loss: Vol I: Attachment.* Harmonsworth: Penguin.

[B7] Bowlby J. (1973). *Attachment and loss: Vol I: Separation.* New York, NY: Basic Books.

[B8] Brand M. Wegmann E. Stark R. Müller A. Wölfling K. Robbins T. W. (2019). The Interaction of Person-Affect-Cognition-Execution (I-PACE) model for addictive behaviors: update, generalization to addictive behaviors beyond internet-use disorders, and specification of the process character of addictive behaviors. *Neurosci. Biobehav. Rev*. 104 1–10. 10.1016/j.neubiorev.2019.06.032 31247240

[B9] Browne M. W. Cudeck R. (1989). Single sample cross-validation indices for covariance structures. *Multivariate Behav. Res.* 24 445–455. 10.1207/s15327906mbr2404_4 26753509

[B10] Busch P. A. McCarthy S. (2021). Antecedents and consequences of problematic smartphone use: a systematic literature review of an emerging research area. *Comput. Hum. Behav.* 114:106414. 10.1016/j.chb.2020.106414

[B11] Chatterjee S. Chaudhuri R. Vrontis D. (2022). Examining the antecedents and consequences of addiction to mobile games: an empirical study. *Inf. Syst. E-Bus. Manage.* 1–20. 10.1007/s10257-022-00614-y

[B12] Chen A. (2019). From attachment to addiction: the mediating role of need satisfaction on social networking sites. *Comput. Hum. Behav.* 98 80–92. 10.1016/j.chb.2019.03.034

[B13] Chen Y. Li R. Liu X. (2023). Problematic smartphone usage among Chinese adolescents: role of social/non-social loneliness, use motivations, and grade difference. *Curr. Psychol.* 42 11529–11538. 10.1007/s12144-021-02458-0

[B14] Dalbudak E. Evren C. Aldemir S. Coskun K. S. Ugurlu H. Yildirim F. G. (2013). Relationship of internet addiction severity with depression, anxiety, and alexithymia, temperament and character in university students. *Cyberpsychol. Behav. Soc. Netw.* 16 272–278. 10.1089/cyber.2012.0390 23363230

[B15] Ding Y. Huang H. Zhang Y. Peng Q. Yu J. Lu G. (2022). Correlations between smartphone addiction and alexithymia, attachment style, and subjective well-being: a meta-analysis. *Front. Psychol.* 13:971735. 10.3389/fpsyg.2022.971735 36124050 PMC9481561

[B16] Dong L. Ren Y. Peng Y. Zhou S. (2017). Development of a motivation for smartphone use scale for adolescents. *J. Clin. Psychol.* 25 863–867. 10.16128/j.cnki.1005-3611.2017.05.016

[B17] Eighmey J. McCord L. (1998). Adding value in the information age: uses and gratifications of sites on the World Wide Web. *J. Bus. Res.* 41 187–194. 10.1016/S0148-2963(97)00061-1

[B18] Fraley R. C. Heffernan M. E. Vicary A. M. Brumbaugh C. C. (2011). The experiences in close relationships-relationship structures questionnaire: a method for assessing attachment orientations across relationships. *Psychol. Assess.* 23 615–625. 10.1037/a0022898 21443364

[B19] Fu X. Liu J. Liu R. D. Ding Y. Wang J. Zhen R. (2020). Parental monitoring and adolescent problematic mobile phone use: the mediating role of escape motivation and the moderating role of shyness. *Int. J. Environ. Health. Res.* 17 1–15. 10.3390/ijerph17051487 32106623 PMC7084728

[B20] Gao Q. Zheng H. Sun R. Lu S. (2022). Parent-adolescent relationships, peer relationships, and adolescent mobile phone addiction: the mediating role of psychological needs satisfaction. *Addict. Behav.* 129:107260. 10.1016/j.addbeh.2022.107260 35151093

[B21] Gao T. Li J. Zhang H. Gao J. Kong Y. Hu Y. (2018). The influence of alexithymia on mobile phone addiction: the role of depression, anxiety and stress. *J. Affect. Disord.* 225 761–766. 10.1016/j.jad.2017.08.020 28926906

[B22] Ge J. Liu Y. Cao W. Zhou S. (2022). The relationship between anxiety and depression with smartphone addiction among college students: the mediating effect of executive dysfunction. *Front. Psychol.* 13:1033304. 10.3389/fpsyg.2022.1033304 36710811 PMC9874858

[B23] Ghinassi S. Casale S. (2023). The role of attachment in gambling behaviors and gambling disorder: A systematic review. *J. Gambl. Stud*. 39, 713–749. 10.1007/s10899-022-10163-1 36322300 PMC10175436

[B24] Ghorbani F. Khosravani V. Bastan F. S. Ardakani R. J. (2017). The alexithymia, emotion regulation, emotion regulation difficulties, positive and negative affects, and suicidal risk in alcohol-dependent outpatients. *Psychiatry Res.* 252 223–230. 10.1016/j.psychres.2017.03.005 28285249

[B25] Gündoğmuş İ Aydın M. S. Algül A. (2021). The relationship of smartphone addiction and alexithymia. *Psychiatry Investig.* 18 841–849. 10.30773/pi.2021.0072 34517444 PMC8473865

[B26] Hao Z. Jin L. (2020). Alexithymia and problematic mobile phone use: a moderated mediation model. *Front. Psychol.* 11:541507. 10.3389/fpsyg.2020.541507 33041910 PMC7522167

[B27] Hassen N. B. Molins F. Garrote-Petisco D. Serrano M. Á (2023). Emotional regulation deficits in autism spectrum disorder: the role of alexithymia and interoception. *Res. Dev. Disabil.* 132:104378. 10.1016/j.ridd.2022.104378 36410287

[B28] Huang S. Lai X. Li Y. Luo Y. Wang Y. (2021). Understanding juveniles’ problematic smartphone use and related influencing factors: a network perspective. *J. Behav. Addict.* 10 811–826. 10.1556/2006.2021.00048 34406975 PMC8997212

[B29] Jauregui P. Estevez A. (2020). Predictive role of attachment, coping, and emotion regulation in gambling motives of adolescents and young people. *J. Gambl. Stud.* 36 1283–1300. 10.1007/s10899-019-09893-6 31535265

[B30] Kim E. Koh E. (2018). Avoidant attachment and smartphone addiction in college students: the mediating effects of anxiety and self-esteem. *Comput. Hum. Behav.* 84 264–271. 10.1016/j.chb.2018.02.037

[B31] Kim E. Cho I. Kim E. (2017). Structural equation model of smartphone addiction based on adult attachment theory: mediating effects of loneliness and depression. *Asian. Nurs. Res.* 11 92–97. 10.1016/j.anr.2017.05.002 28688505

[B32] Kline T. J. (2005). *Psychological testing: a practical approach to design and evaluation.* Thousand Oaks, CA: Sage Publications.

[B33] Lai X. Huang S. Nie C. Yan J. J. Li Y. Wang Y. (2022). Trajectory of problematic smartphone use among adolescents aged 10-18 years: the roles of childhood family environment and concurrent parent-child relationships. *J. Behav. Addict.* 11 577–587. 10.1556/2006.2022.00047 35895472 PMC9295210

[B34] Leung L. Wei R. (2000). More than just talk on the move: uses and gratifications of the cellular phone. *Journal. Mass. Commun. Q.* 77 308–320. 10.1177/10776990000770020

[B35] Li J. Zhan D. Zhou Y. Gao X. (2021). Loneliness and problematic mobile phone use among adolescents during the COVID-19 pandemic: the roles of escape motivation and self-control. *Addict. Behav.* 118 1–7. 10.1016/j.addbeh.2021.106857 33676160 PMC8598166

[B36] Li Z. K. Shi L. J. Cai X. L. (2022). Smartphone addiction is more harmful to adolescents than Internet gaming disorder: divergence in the impact of parenting styles. *Front. Psychol.* 13:1044190. 10.3389/fpsyg.2022.1044190 36591056 PMC9796998

[B37] Liese B. S. Kim H. S. Hodgins D. C. (2020). Insecure attachment and addiction: testing the mediating role of emotion dysregulation in four potentially addictive behaviors. *Addict. Behav.* 107:106432. 10.1016/j.addbeh.2020.106432 32330652

[B38] Lin Y. H. Pan Y. C. Lin S. H. Chen S. H. (2017). Development of short-form and screening cutoff point of the Smartphone Addiction Inventory (SPAI-SF). *Int. J. Methods Psychiatr. Res.* 26 1–6. 10.1002/mpr.1525 27658956 PMC6877212

[B39] Liu C. Ma J. L. (2019). Adult attachment style, emotion regulation, and social networking sites addiction. *Front. Psychol.* 10:02352. 10.3389/fpsyg.2019.02352 31749729 PMC6843004

[B40] Liu Q. Q. Yang X. J. Hu Y. T. Zhang C. Y. Nie Y. G. (2020). How and when is family dysfunction associated with adolescent mobile phone addiction? testing a moderated mediation model. *Child. Youth Serv. Rev.* 111:104827. 10.1016/j.childyouth.2020.104827

[B41] Lyvers M. Ryan N. Thorberg F. A. (2022). Alexithymia, attachment security and negative mood. *Aust. Psychol*. 57 86–94. 10.1080/00050067.2022.2045173

[B42] Musetti A. Manari T. Billieux J. Starcevic V. Schimmenti A. (2022). Problematic social networking sites use and attachment: a systematic review. *Comput. Hum. Behav.* 131:107199. 10.1016/j.chb.2022.107199

[B43] Ng C. S. Chan V. C. (2020). Prevalence and associated factors of alexithymia among Chinese adolescents in Hong Kong. *Psychiatry. Res.* 290:113126. 10.1016/j.psychres.2020.113126 32505928

[B44] Nigro G. Cosenza M. Ciccarelli M. (2017). The blurred future of adolescent gamblers: impulsivity, time horizon, and emotional distress. *Front. Psychol.* 8:486. 10.3389/fpsyg.2017.00486 28421013 PMC5376625

[B45] Pellerone M. Tomasello G. Migliorisi S. (2017). Relationship between parenting, alexithymia and adult attachment styles: a cross-sectional study on a group of adolescents and young adults. *Clin. Neuropsychiatr.* 14 125–134.

[B46] Ridout N. Smith J. Hawkins H. (2021). The influence of alexithymia on memory for emotional faces and realistic social interactions. *Cogn. Emot.* 35 540–558. 10.1080/02699931.2020.1747991 32268841

[B47] Sanchez G. (2013). *Pls path modeling with R.* Berkeley: Trowchez Editions.

[B48] Schaller M. Kenrick D. T. Neel R. Neuberg S. L. (2017). Evolution and human motivation: a fundamental motives framework. *Soc. Personal. Psychol. Compass.* 11 1–15. 10.1111/spc3.12319

[B49] Schreiber J. B. Nora A. Stage F. K. Barlow E. A. King J. (2006). Reporting structural equation modeling and confirmatory factor analysis results: a review. *J. Educ. Res.* 99 323–338. 10.3200/JOER.99.6.323-338

[B50] Scigala D. K. Fabris M. A. Badenes-Ribera L. Zdankiewicz-Scigala E. Hintertan I. Longobardi C. (2022). Alexithymia and adult attachment: investigating the mediating role of fear of intimacy and negative mood regulation expectancies. *Psychol. Rep.* 125 1896–1914. 10.1177/00332941211010252 33878970

[B51] Scigala D. K. Fabris M. A. Badenes-Ribera L. Zdankiewicz-Scigala E. Longobardi C. (2021). Alexithymia and self differentiation: the role of fear of intimacy and insecure adult attachment. *Contemp. Fam. Ther.* 43 165–176. 10.1007/s10591-021-09567-9

[B52] Shutzman B. Gershy N. (2023). Children’s excessive digital media use, mental health problems and the protective role of parenting during COVID-19. *Comput. Hum. Behav.* 139:107559. 10.1016/j.chb.2022.107559 36405875 PMC9650221

[B53] Sifneos P. E. (1973). The prevalence of ‘alexithymic’ characteristics in psychosomatic patients. *Psychother. Psychosom.* 22 255–262. 10.1159/000286529 4770536

[B54] Sutherland B. D. Fallah-Sohy N. Kopera M. Jakubczyk A. Sutherland M. T. Trucco E. M. (2022). Alexithymia mediates the association between childhood trauma and adolescent E-cigarette use. *Drug. Alcohol. Depend.* 236:109500. 10.1016/j.drugalcdep.2022.109500 35623159 PMC9384700

[B55] Tarantino S. Papetti L. De Ranieri C. Boldrini F. Rocco A. M. D’Ambrosio M. (2018). Maternal alexithymia and attachment style: which relationship with their children’s headache features and psychological profile? *Front. Neurol.* 8:751. 10.3389/fneur.2017.00751 29403425 PMC5786507

[B56] Wen F. Ding Y. Yang C. Ma S. Zhu J. Xiao H. (2022). Influence of smartphone use motives on smartphone addiction during the COVID-19 epidemic in China: the moderating effect of age. *Curr. Psychol.* 10.1007/s12144-022-03355-w [Epub ahead of print]. 35854703 PMC9282147

[B57] Wolniewicz C. A. Rozgonjuk D. Elhai J. D. (2020). Boredom proneness and fear of missing out mediate relations between depression and anxiety with problematic smartphone use. *Hum. Behav. Emerg. Techno.* 2 61–70. 10.1002/hbe2.159

[B58] Xiang Y. He Q. Yuan R. (2022). Childhood maltreatment affects mobile phone addiction from the perspective of attachment theory. *Int. J. Ment. Health Addict.* 1–13. 10.1007/s11469-022-00806-0

[B59] Xiao W. Zhou H. Li X. Lin X. (2021). Why are individuals with alexithymia symptoms more likely to have mobile phone addiction? The multiple mediating roles of social interaction anxiousness and boredom proneness. *Psychol. Res. Behav. Manag.* 14 1631–1641. 10.2147/PRBM.S328768 34675703 PMC8518138

[B60] Yuchang J. Cuicui S. Junxiu A. Junyi L. (2017). Attachment styles and smartphone addiction in Chinese college students: the mediating roles of dysfunctional attitudes and self-esteem. *Int. J. Ment. Health Addict.* 15 1122–1134. 10.1007/s11469-017-9772-9

[B61] Yuchang J. Junyi L. Junxiu A. Jing W. Mingcheng H. (2019). The differential victimization associated with depression and anxiety in cross-cultural perspective: a meta-analysis. *Trauma Violence Abus*. 20 560–573. 10.1177/152483801772642 29333963

[B62] Zhang M. X. Wu A. M. (2022). Effects of childhood adversity on smartphone addiction: the multiple mediation of life history strategies and smartphone use motivations. *Comput. Hum. Behav.* 134:107298. 10.1016/j.chb.2022.107298

[B63] Zhang M. X. Zhou H. Yang H. M. Wu A. M. (2023). The prospective effect of problematic smartphone use and fear of missing out on sleep among Chinese adolescents. *Curr. Psychol*. 42 5297–5305. 10.1007/s12144-021-01863-9

[B64] Zhang Q. Hou Z. J. Fraley R. C. Hu Y. Zhang X. Zhang J. (2022). Validating the experiences in close relationships-relationship structures scale among Chinese children and adolescents. *J. Pers. Assess.* 104 347–358. 10.1080/00223891.2021.1947844 34292844

[B65] Zhou H. Xiao W. Li X. Jiang H. (2022). The influence of alexithymia on problematic mobile phone use among Chinese adolescent students: multiple mediating roles of social interaction anxiousness and core self-evaluations. *J. Affect. Disord.* 308 569–576. 10.1016/j.jad.2022.04.051 35429535

[B66] Zhu X. Yi J. Yao S. Ryder A. G. Taylor G. J. Bagby R. M. (2007). Cross-cultural validation of a Chinese translation of the 20-item Toronto Alexithymia Scale. *Compr. Psychiatry* 48 489–496. 10.1016/j.comppsych.2007.04.007 17707259

