# Supplementary material for: Effects of use motivations and alexithymia on smartphone addiction: mediating role of insecure attachment
Source: Front Psychol. 2023 Jul 17;14:1227931. doi: 10.3389/fpsyg.2023.1227931 (PMC10389275; doi:10.3389/fpsyg.2023.1227931)
Supplement: Supplementary file 1 [file Table_1.docx]

# Supplementary Material

# Table S1

TABLE S1. Results of EFA and CFA

| **Constructs** | **Items** | **Mean** | **SD** | **FFA Factor Loading** | **CFA Std. Estimate** |
| --- | --- | --- | --- | --- | --- |
| Smartphone addiction  (SA) | SA1 | 2.100 | 0.858 | 0.756 | 0.700 |
|  | SA2 | 3.727 | 1.814 | 0.733 | 0.625 |
|  | SA3 | 2.235 | 0.832 | 0.710 | 0.741 |
|  | SA4 | 2.338 | 0.826 | 0.702 | 0.731 |
|  | SA5 | 4.626 | 1.628 | 0.669 | 0.732 |
|  | SA6 | 2.570 | 0.826 | 0.697 | 0.755 |
|  | SA7 | 4.057 | 1.855 | 0.685 | 0.708 |
|  | SA8 | 4.437 | 1.778 | 0.659 | 0.723 |
|  | SA9 | 2.500 | 0.794 | 0.703 | 0.745 |
|  | SA10 | 2.229 | 0.766 | 0.690 | 0.679 |
| Difficulty identifying feelings  (DIF) | DIF1 | 2.977 | 1.065 | 0.766 | 0.732 |
|  | DIF2 | 2.940 | 1.067 | 0.829 | 0.828 |
|  | DIF3 | 3.139 | 1.035 | 0.777 | 0.751 |
|  | DIF4 | 2.916 | 1.023 | 0.782 | 0.763 |
|  | DIF5 | 3.000 | 1.027 | 0.764 | 0.729 |
|  | DIF6 | 3.146 | 0.970 | 0.760 | 0.736 |
|  | DIF7 | 3.071 | 0.999 | 0.741 | 0.726 |
| Difficulty describing feelings  (DDF) | DDF 1 | 3.461 | 0.976 | 0.755 | 0.567 |
|  | DDF 2 | 3.311 | 0.970 | 0.755 | 0.563 |
|  | DDF 3 | 3.143 | 1.024 | 0.766 | 0.694 |
|  | DDF 4 | 3.021 | 1.070 | 0.825 | 0.898 |
|  | DDF 5 | 3.059 | 1.027 | 0.801 | 0.873 |
| Externally oriented thinking  (EOT) | EOT 1 | 2.932 | 0.95 | 0.778 | 0.788 |
|  | EOT 2 | 2.829 | 0.965 | 0.787 | 0.758 |
|  | EOT 3 | 3.012 | 1.044 | 0.763 | 0.765 |
|  | EOT 4 | 2.975 | 0.986 | 0.771 | 0.781 |
|  | EOT 5 | 2.846 | 0.972 | 0.734 | 0.722 |
|  | EOT 6 | 2.922 | 0.944 | 0.742 | 0.721 |
|  | EOT 7 | 2.880 | 0.967 | 0.784 | 0.798 |
|  | EOT 8 | 3.028 | 0.946 | 0.724 | 0.702 |
| Social motivation (SM) | SM1 | 4.179 | 1.686 | 0.821 | 0.838 |
|  | SM2 | 4.418 | 1.741 | 0.802 | 0.803 |
|  | SM5 | 4.570 | 1.655 | 0.763 | 0.794 |
| Escape motivation (EM) | EM1 | 5.690 | 1.182 | 0.880 | 0.829 |
|  | EM4 | 5.679 | 1.191 | 0.887 | 0.879 |
|  | EM5 | 5.537 | 1.256 | 0.848 | 0.796 |
| Attachment avoidance  (AAV) | AAV1 | 3.441 | 1.584 | 0.799 | 0.883 |
|  | AAV2 | 3.497 | 1.656 | 0.861 | 0.682 |
|  | AAV3 | 3.560 | 1.651 | 0.790 | 0.93 |
|  | AAV4 | 3.845 | 1.588 | 0.719 | 0.776 |
|  | AAV5 | 3.713 | 1.696 | 0.773 | 0.634 |
|  | AAV6 | 3.574 | 1.567 | 0.774 | 0.724 |
| Attachment anxiety  (AAN) | AAN1 | 3.751 | 1.704 | 0.846 | 0.778 |
|  | AAN2 | 4.295 | 1.662 | 0.898 | 0.917 |
|  | AAN4 | 4.433 | 1.652 | 0.837 | 0.796 |
